# *Cutibacteriumacnes* Septic Arthritis of the Nonoperated Knee: A Case Report

**DOI:** 10.1055/s-0037-1603970

**Published:** 2017-07-05

**Authors:** Kelechi R. Okoroha, Michael D. Gabbard, Jamal Fitts, Trevor R. Banka

**Affiliations:** 1 Department of Orthopedic Surgery, Henry Ford Hospital, Detroit, Michigan; 2 Morehouse School of Medicine, Atlanta, Georgia

**Keywords:** *Propionibacteriumacnes*, *Cutibacteriumacnes*, septic arthritis, intra-articular injection, steroid injection, septic knee, corticosteroid

## Abstract

*Cutibacterium (Propionibacterium) acnes* , a gram-positive bacillus with low pathogenicity, is an uncommon but known cause of prosthetic joint infections, particularly related to shoulder surgery. *C. acnes* , however, is an extremely rare pathogen in the nonoperated knee joint. This report details an uncommon case of *C. acnes* septic knee arthritis after multiple intra-articular steroid injections in a 56-year-old male patient. After an indolent presentation and late diagnosis, the patient underwent surgical debridement with IV antibiotic management. This case illustrates that intra-articular corticosteroid injections for the management of osteoarthritis are not without risk. Literature supporting their use remains limited and clinicians should use proficient clinical judgment for appropriate patient selection for these injections. Vigilance following injections or aspirations of the knee should be maintained to identify the indolent clinical presentation of *C. acnes* septic arthritis.

Intra-articular injections are a common practice in conservative management of knee osteoarthritis. 1 Septic arthritis following these injections is a rare but potentially devastating complication. 2 The culpable microorganisms in the majority of cases are from the *Staphylococcus or Streptococcus sp* . 3 Septic arthritis of the nonoperated knee joint caused by *Cutibacterium* ( *Propionibacterium) acnes* is not often expected or encountered. The more common presentation of this pathogen in orthopaedics is an indolent prosthetic joint or spinal infection. We present a rare case of *C. acnes* septic arthritis of the nonoperated knee, following multiple intra-articular injections. This case demonstrates the subtle presentation of *C. acnes* septic arthritis and suggests careful consideration of the implications of intra-articular injections in management of knee osteoarthritis.

## Case Report

A 56-year-old male patient with a medical history of diabetes (HgA1c, 7.5) and obesity (body mass index [BMI], 39.15) presented to a sports medicine clinic complaining of right knee pain and mechanical symptoms. Radiographic and clinical evaluation was consistent with primary osteoarthritis. His symptoms were treated conservatively with physical therapy, nonsteroidal anti-inflammatory drugs (NSAIDs), sodium hyaluronate injections, and multiple corticosteroid injections.

The patient returned a year later with acute exacerbation of chronic knee pain. An aspiration was performed under sterile technique and the analysis demonstrated 68,000 white blood cells (WBCs), 88% polymorphonuclear (PMN) leukocytes, positive uric acid crystals, and negative cultures. Indomethacin and colchicine were initiated to treat gout with transient symptomatic improvement. At 1-week follow-up, the patient presented with recurrent pain accompanied by erythema and swelling. Due to concern for septic arthritis, the patient was admitted to the hospital. Aspiration was attempted and he was initiated on IV vancomycin for cellulitis. Gout treatment was continued and he was discharged on oral antibiotics following significant symptomatic improvement.

Two weeks later, the patient followed up with continued knee pain. Due to concern for low-grade infection, arthrocentesis was performed under sterile technique. Analysis of the fluid demonstrated 81,000 WBCs, 92% PMN leukocytes with uric acid crystals. Cultures were initially negative. However, 2 weeks later, the patient presented on referral to the senior author (T.B.) where cultures were discovered to be positive for *C. acnes* . A new aspiration was obtained. The Gram stain was positive for the bacillus; however, cultures were held for 2 weeks and no microorganisms were isolated.

Following these negative cultures, a confirmatory aspiration was repeated due to result inconsistency. The Gram stain showed gram-positive bacilli, and it was determined that the patient had a chronic *C. acnes* septic arthritis. The patient elected to undergo surgical irrigation and debridement. Intraoperatively, inflamed synovium was noted without gross purulence. Tissue and synovial fluid samples were taken and later cultured for *C. acnes* . The patient received IV ceftriaxone therapy for 6 weeks and oral moxifloxacin for an additional 2 weeks.

After irrigation and debridement, the patient had significant improvement in pain and function. At 1-year postoperative, the patient had continued knee pain with radiographic evidence of osteoarthritis. His inflammatory markers returned to normal and there were no further signs of infection. Joint replacement was postponed due to concern of potential for prosthetic joint infection.

## Discussion

*C. acnes* is a nonspore forming, anaerobic, aerotolerant, gram-positive bacillus with low pathogenicity. The bacterium is a normal inhabitant of hair follicles and sebaceous glands of the skin. 4 *C. acnes* is a known cause of implant-associated infections, most commonly in the shoulder. The increased abundance of *C. acnes* in the upper parts of the body may explain the higher prevalence of infections associated with the shoulder. The pathogenesis of joint infections is related to the microorganism's multiple resistance mechanisms including biofilm formation. 4 *C. acnes* may be under diagnosed, as culture growth occurs after the typical 5-day incubation period. Physicians must request cultures to be held for an extended time, as it may take up to 21 days for *C. acnes* to grow on culture medium. 5 In our case, cultures were initially negative, but were subsequently found to be positive when rechecked at a later date. Interestingly, multiple Gram stains demonstrated gram-positive bacilli without culture growth. Due to these discrepancies, we now routinely hold cultures for an extended duration and communicate suspicion of *C. acnes.*

Intra-articular injections are commonly used in the management of symptomatic osteoarthritis. In 2006, a Cochrane review was performed on treatment of knee arthritis using intra-articular steroids. 1 The review supported the role of intra-articular steroids for knee arthritis due to demonstration of a brief but significant improvement in pain compared with placebo. The 2013 American Academy of Orthopaedic Surgeons (AAOS) guidelines found limited literature demonstrating benefit of intra-articular steroids compared with placebo and found no improvement with hyaluronic injections. The academy was inconclusive in their recommendation on the role of intra-articular corticosteroids and recommended against intra-articular hyaluronic acid for treatment of osteoarthritis of the knee. 6 Currently at our institution, hyaluronic acid injections are generally not performed and corticosteroid injections are used sparingly for acute exacerbations of arthritic pain.

Intra-articular injections are generally believed to be safe with only mild side effects. One rare but potentially devastating complication is septic arthritis. Its incidence varies from 1:3,000 to 1:50,000. 2 Shemesh et al reported a case series of six patients, with a mean age of 75, who developed staphylococcal or streptococcal septic arthritis within 1 week of an intra-articular knee injection. 7 In their report, all patients had multiple medical comorbidities with 3 patients receiving corticosteroid injections and 3 hyaluronic acid injections. One patient ultimately succumbed to complications of septic shock. Traditional presentation of septic arthritis includes pain, swelling, difficulty with ambulation, and limited range of motion. However, a subtle presentation may occur with infection of *C. acnes* . Underlying osteoarthritis or the effects of steroid injections may mask the infection. Our patient demonstrates the indolent presentation of *C. acnes* and shows that infection can be present without gross purulence during surgical debridement.

It has been shown in previous trials that acute infections may be more common in Type II diabetic patients than age-matched nondiabetic patients. 8 Our patient in this case also had diabetes, which was treated by medication. The patient's most recent HgbA1c before the infection was shown to be slightly elevated. One could suspect that his diabetes also could have played a role in acquiring septic arthritis after the injection. Diabetes is a common comorbidity of individuals who receive intra-articular steroid injections. Tighter controls of diabetes may need to be required before performing even minimally invasive procedures, especially in those with poorly controlled diabetes. There must be a low threshold for suspicion of infection in this patient population.

Obesity has a clear but not yet precisely defined effect on the immune response through a variety of immune mediators that lead to susceptibility to infections. 9 Our patient was classified as class II obesity due to his BMI. It is likely that the patient's obesity could influence his infection by an unusual pathogen as well. There is little literature on the bacterial flora around the knee of a severely obese patient, and may serve as a subject that should be further investigated in the future.

Dubost et al reviewed responsible microorganisms for septic arthritis in a single center over 3 decades. They noted *Staphylococcus* species to be responsible for two-thirds of 374 cases and nonidentified anaerobic bacteria to be responsible for 2/374 cases. 3 Previous studies by Kooijmans-Coutinho et al and Yocum et al describe a total of three cases of *C. acnes* septic knee arthritis. 10 11 One case presented due to hematogenous seeding in a 71-year-old male patient with polyarthralgia and no previous history of injections. The other two reports describe female patients with rheumatoid arthritis who developed *C. acnes* septic arthritis; one following a corticosteroid injection and the other an arthrocentesis. The patients had similar presentations; both were afebrile, had increased inflammatory markers, and persistent knee pain and swelling. Both patients were treated nonoperatively with antibiotics and had good clinical outcomes. These cases are similar to our patient in that they all had indolent presentations. Our case differed in that our patients' symptoms failed nonoperative management and required operative debridement.

In conclusion, the case presented highlights a rare case of septic arthritis in the nonoperated knee due to the anaerobic bacterium, *C. acnes,* following multiple intra-articular injections. Intra-articular corticosteroids have long been a mainstay of osteoarthritis treatment. However, literature is still scarce to support their long-term benefits. Although risks of these injections appear minimal, there have been multiple reports of septic arthritis following injections. This highlights the importance of critical analysis of patient factors prior to utilizing these injections in the treatment of osteoarthritis and brings awareness to the possibility of the devastating complication of septic arthritis. Clinicians should remain vigilant in identifying possible cases of septic arthritis following intra-articular corticosteroid injections. In particular, awareness of the unique presentation of *C. acnes* and the need to maintain cultures for longer duration to identify this uncommon microorganism can prevent misdiagnosis and its associated morbidity. 12
